# Hemodynamic Study of Flow Remodeling Stent Graft for the Treatment of Highly Angulated Abdominal Aortic Aneurysm

**DOI:** 10.1155/2016/3830123

**Published:** 2016-05-09

**Authors:** Siang Lin Yeow, Hwa Liang Leo

**Affiliations:** Department of Biomedical Engineering, Faculty of Engineering, National University of Singapore, Singapore 117575

## Abstract

This study investigates the effect of a novel flow remodeling stent graft (FRSG) on the hemodynamic characteristics in highly angulated abdominal aortic aneurysm based on computational fluid dynamics (CFD) approach. An idealized aortic aneurysm with varying aortic neck angulations was constructed and CFD simulations were performed on nonstented models and stented models with FRSG. The influence of FRSG intervention on the hemodynamic performance is analyzed and compared in terms of flow patterns, wall shear stress (WSS), and pressure distribution in the aneurysm. The findings showed that aortic neck angulations significantly influence the velocity flow field in nonstented models, with larger angulations shifting the mainstream blood flow towards the center of the aorta. By introducing FRSG treatment into the aneurysm, erratic flow recirculation pattern in the aneurysm sac diminishes while the average velocity magnitude in the aneurysm sac was reduced in the range of 39% to 53%. FRSG intervention protects the aneurysm against the impacts of high velocity concentrated flow and decreases wall shear stress by more than 50%. The simulation results highlighted that FRSG may effectively treat aneurysm with high aortic neck angulations via the mechanism of promoting thrombus formation and subsequently led to the resorption of the aneurysm.

## 1. Introduction

Aortic aneurysms are dilation of the aorta commonly found at the thoracic, abdominal, and thoracoabdominal regions. Generally, if left untreated, aneurysm rupture carries a risk of death up to 90% [1]. Endovascular aortic aneurysm repair (EVAR) is a minimally invasive surgery that has become the treatment of choice for most eligible patients due to lower mortality, lower perioperative morbidity risk, and similar long-term survival to open surgery [2, 3]. However, not every patient can receive EVAR treatment. Nearly 35% of men and 60% of women are ineligible for EVAR due to complex aneurysm morphology such as short aortic neck and highly angulated aortic neck [4].

Recently, new endovascular strategies based on flow diversion concept in treating aortic aneurysm have emerged [5–7]. Conventional stent graft functions as a mechanical barrier by excluding blood flow into the aneurysm. Unlike conventional stent graft, the flow diversion concept acts as a passive barrier by reducing blood flow into the aneurysm that will subsequently create a natural hemodynamic environment to promote aneurysm thrombosis [8]. The flow diversion concept creates hemodynamic effects that are conducive for the activation and aggregation of platelets within the aneurysm sac [9]. Subsequently, the mechanism is postulated to promote thrombus formation that will eventually lead to degradation of the aneurysm [10].

At the moment, two types of flow diversion solutions are used in clinical settings to treat aortic aneurysms. The first concept of flow diversion is a commercial multilayer flow-modulator (MFM) stent (Cardiatis, Isnes, Belgium) that has been used to treat arterial aneurysms in situation whereby patients were unfit for standard endovascular device intervention. However, MFM stent is approved in limited countries, costly, and technically demanding [11]. An alternative strategy has been adopted by clinicians using multiple overlapping uncovered stent (MOUS) to achieve similar flow diversion effect [11–13]. Short-term clinical outcome using MOUS for treatment of complicated aortic aneurysm was encouraging in small group of patients, thus requiring further investigation before wide clinical adoption.

Although short-term clinical outcome has been encouraging, the actual understanding of flow diversion mechanism in aortic aneurysm is still in its infancy and warrants further investigation. Recent hemodynamic studies on MFM and MOUS have shown reduced blood velocity near the aneurysm wall with significant decrease in wall shear stress and exhibited more uniform pressure distribution acting on the aneurysm wall [14–16]. At the moment, there are no studies of flow diversion devices on patients with complex aortic aneurysm such as high-angulated neck whereby aortic neck angle significantly influences the blood flow patterns [17]. Therefore, to further understand the efficacy of flow diversion concept, the hemodynamic effects in complex aortic aneurysm before and after device intervention are worth studying.

In this study, the authors present an alternative flow diversion concept with a novel flow remodeling stent graft (FRSG). The proposed FRSG is based on covered stent graft with porous opening on its surface. FRSG could achieve the same flow diversion effect as MFM and MOUS through flow remodeling in the aneurysm, thus providing an environment that is favorable to thrombus formation and eventually leads to the degradation of aneurysm. The present study aimed to investigate the effect of hemodynamic performance based on the newly proposed flow remodeling stent graft on four severely angulated aortic necks. Idealized abdominal aortic aneurysm models with a variation of aortic neck angulations were constructed. Computational fluid dynamics (CFD) method is used to investigate the distribution flow patterns, wall shear stress, and pressure along the aneurysm wall. The difference between nonstented and stented models is discussed.

## 2. Methods

### 2.1. Geometry

**Figure 1:**
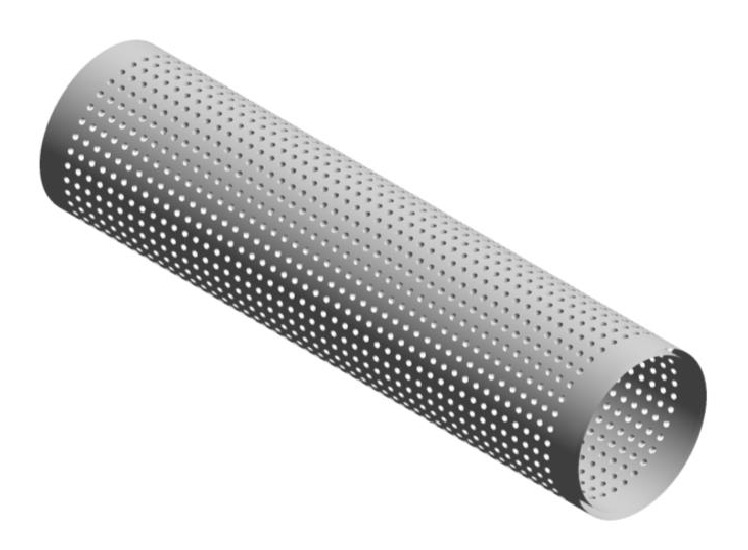
Modeled flow remodeling stent graft (FRSG).

The flow remodeling stent graft (FRSG) is a covered stent graft with circular pores as shown in Figure 1. The proximal and distal zones are covered ensuring adequate seal zone to prevent type 1 endoleak. The stent graft is 25 mm in diameter and has a total length of 100 mm. The pore size in the FRSG model was designed with 1 mm in diameter together with both horizontal and longitudinal spacing of 1 mm, respectively.

Idealized fusiform abdominal aortic aneurysm models with a diameter of 5.8 cm were constructed using computer-aided software SolidWorks (Dassault Systèmes SolidWorks Corp., Concord, MA, USA). The aneurysm size chosen in this study is categorized as an average threshold for repair with an average risk of rupture. The neck of the aortic aneurysm was parameterized with an increment of 10 degrees starting from 60 degrees up to 90 degrees to mimic highly angulated aortic neck condition.

**Figure 2:**
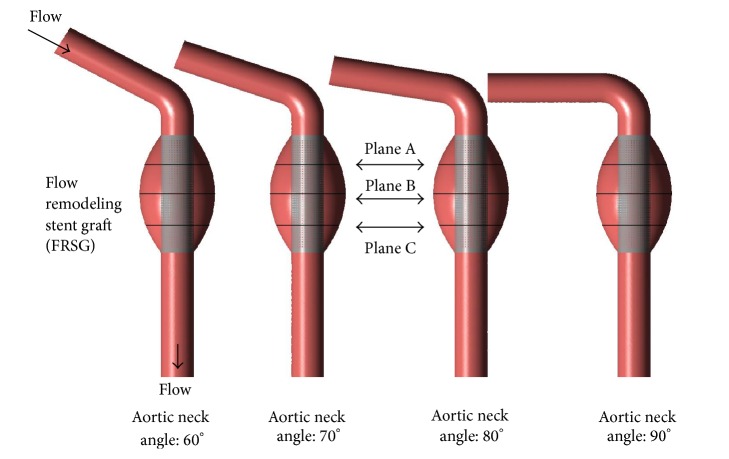
Geometry of FRSG stented aneurysm with varying aortic neck angulation.

The FRSG model was virtually implanted into the four aneurysm models (60°, 70°, 80°, and 90°) using SolidWorks and is shown in Figure 2. Each computational fluid domain was created using ANSYS DesignModeler (ANSYS Inc., Canonsburg, PA).

### 2.2. Meshing

Two sets of computational models, one with nonstented and the other with FRSG of four varying aortic neck angles, were discretized using ANSYS Workbench (ANSYS Inc., Canonsburg, PA). A minimum of 0.25 mm element size was applied to the computational models in both cases. The volume mesh size for nonstented models was 0.67 million hexahedral elements. On the other hand, stented models with FRSG were meshed with tetrahedral elements and yielded 5.8 million elements.

### 2.3. Governing Equations

The Navier-Stokes equations of mass conservation (1) and momentum conservation (2) for incompressible and Newtonian fluid are solved numerically on three-dimensional flow domain:

(1) ∇·u=0,

(2) ρ∂u∂t+ρu·∇u=−∇P+μ∇2u,

where *u*,  *ρ*,  *μ*, and *P* denote velocity, density, dynamic viscosity, and pressure.

### 2.4. Assumptions

The blood flow simulation was performed under steady state condition. Blood was assumed to be incompressible, homogenous, and Newtonian fluid [18]. The Reynolds number (Re) calculated was 757, and the flow was simulated under the laminar flow region [19]. The density and dynamic viscosity were assumed constant with value of 1060 kg/m^3^ and dynamic viscosity of 0.0035 Pa·S, respectively [20].

### 2.5. Boundary Conditions

Rigid and no-slip boundary conditions were imposed on the aortic aneurysm wall and on the FRSG wall. A flat velocity profile with a magnitude of 0.1 m/s is set at the aorta inlet. Meanwhile, a zero diffusion flux boundary condition was applied at the aorta outlet [14].

### 2.6. Numerical Scheme

Steady state simulations of nonstented model and stented model with FRSG within the highly angulated abdominal aortic aneurysm were solved using commercial computational fluid dynamic software, ANSYS FLUENT version 16 (ANSYS Inc., Canonsburg, Pennsylvania, USA). The coupling between pressure field and velocity field is solved using SIMPLE algorithm. Second-order upwind scheme was used as the spatial discretization for the flow governing equations. The convergence criteria were set to 10^−4^ for the normalized continuity and velocity residuals. All simulations were run on a computer workstation with 3.10 GHz quad-core processor with 24 GB RAM.

## 3. Results

### 3.1. Aneurysm Flow Pattern

**Figure 3:**
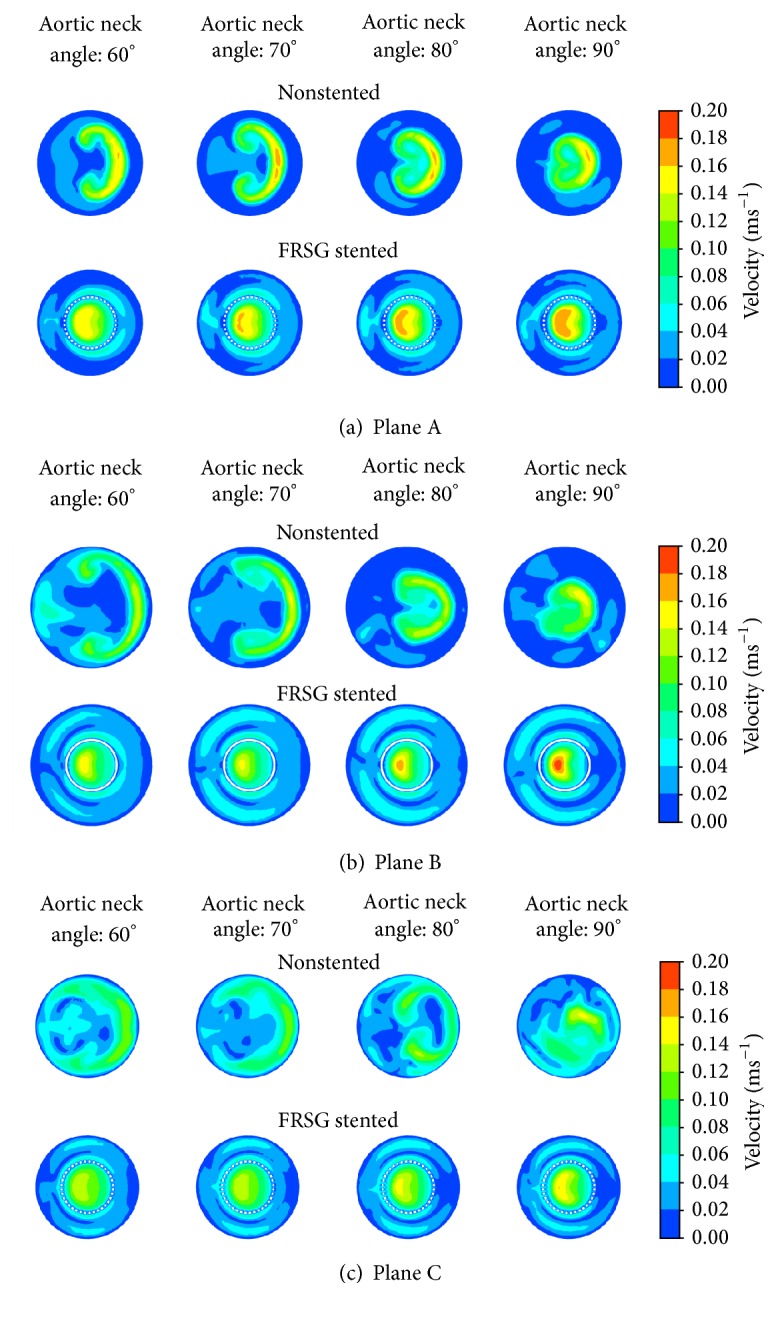
Velocity contours at the horizontal planes of the aneurysm: (a) Plane A located proximal to aneurysm sac, (b) Plane B located at middle of aneurysm sac, and (c) Plane C located distal to aneurysm sac.

**Figure 4:**
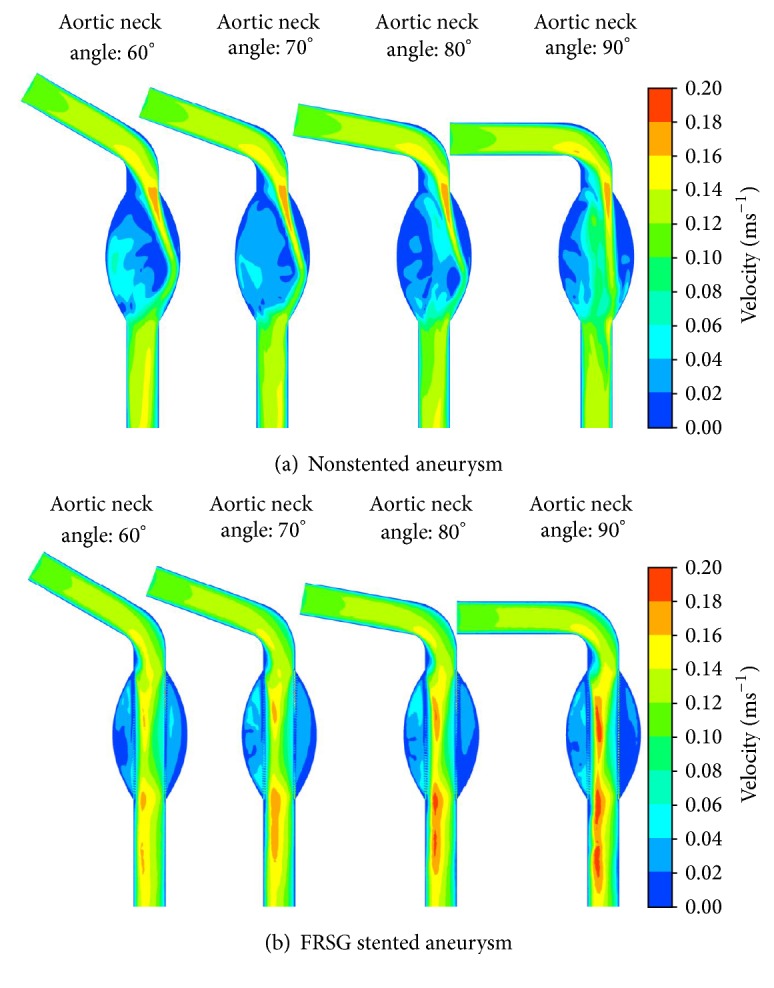
Velocity contours at the longitudinal plane of the aneurysm: (a) velocity contours of nonstented aneurysm and (b) velocity contours of FRSG stented aneurysm.

Detailed flow patterns in the aneurysm sac are presented as velocity contours in three horizontal cross-sectional areas of interest as shown in Figure 3 and one longitudinal cross section as shown in Figure 4. The location of horizontal cross-sectional area can be referred to in Figure 2. Velocity contours at the upper plane of the aneurysm (Plane A) located below the aortic neck are shown in Figure 3(a). The bulk of blood flow forming a crescent-shape velocity contour was observed for nonstented aneurysm (aortic neck angle of 60 degrees and 70 degrees), but as the aortic neck angulation increases, the crescent-shape gradually forms into circular contours located at the center of the aorta. By deploying FSRG into the aneurysm, the crescent-shape velocity contour diminishes and the majority of blood flow was observed to be in the middle of stent graft lumen for all aortic neck angles. Next, Figure 3(b), which represents the flow patterns at the middle plane of the aneurysm sac (Plane B), showed similar transition from crescent-shape velocity contours to circular contours for nonstented aneurysm as the aortic neck angulation increases. Observation revealed that FRSG intervention shifted the bulk flow into the stent graft lumen. Meanwhile, Figure 3(c) shows the velocity contours at the bottom plane of the aneurysm (Plane C). The high velocity regions that are circumferentially located near the nonstented aneurysm wall were effectively dampened by stenting of FRSG.

Next, on the longitudinal cross section, Figure 4(a) highlights a thin strip of high velocity starting from the aneurysm neck towards the distal end of aneurysm sac. With FRSG implanted into the aneurysm, the high velocity regions were found to be isolated in the stent graft lumen thus excluding main bulk of blood flow from the aneurysm sac as shown in Figure 4(b). The flow field distributions were found to be uniform along the outer surface of stent graft in all aneurysms that intervened with FRSG. In Table 1, the average velocity magnitude in FRSG stented aneurysm sac is highlighted as a percentage of the nonstented aneurysm. The average velocity magnitude in aneurysm sac (FRSG stented case) was markedly reduced by 41% and 39% for aortic neck angles of 60 degrees and 70 degrees, respectively. In addition, the average velocity magnitude in FRSG stented aneurysm sac was reduced by more than half (54% and 53%) compared to nonstented aneurysm for aortic neck angles of 80 degrees and 90 degrees, respectively.

**Figure 5:**
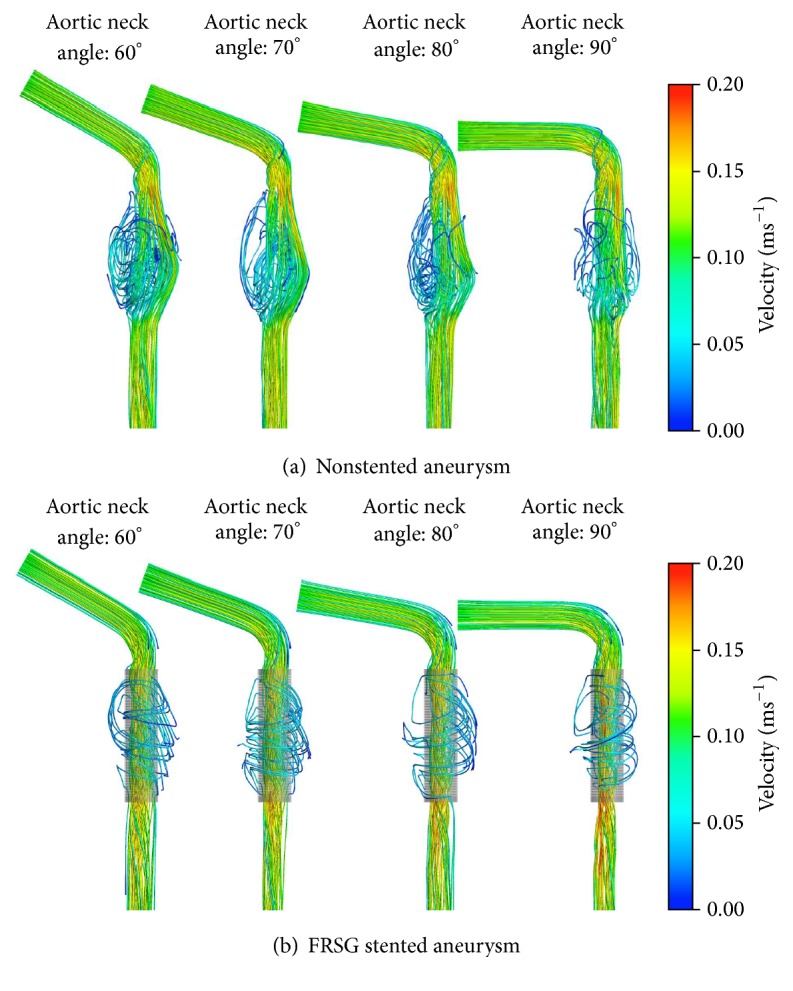
(a) Velocity streamlines of the nonstented aneurysm and (b) velocity streamlines of the FRSG stented aneurysm.

Figure 5(a) shows the velocity streamlines of the nonstented aneurysm models. Distinct recirculation vortex was observed at the aneurysm sac across all aortic neck angulations. However, as the aortic neck angle increases, the vortex pattern in the aneurysm sac was observed to have weakened and the velocity field seemed to be more concentrated towards the center of the aorta. Compared to the stented aneurysm with FRSG as depicted in Figure 5(b), there is strong evidence that the flow pattern was remodeled through the implantation of FRSG. Firstly, less recirculation flows were observed in the aneurysm sac and the bulk of blood flow was seen to shift into the stent lumen. Interestingly, the deployment of FRSG has exhibited laminar flow pattern in stented model in contrast to erratic flow vortex observed in nonstented case.

### 3.2. Wall Shear Stress

**Figure 6:**
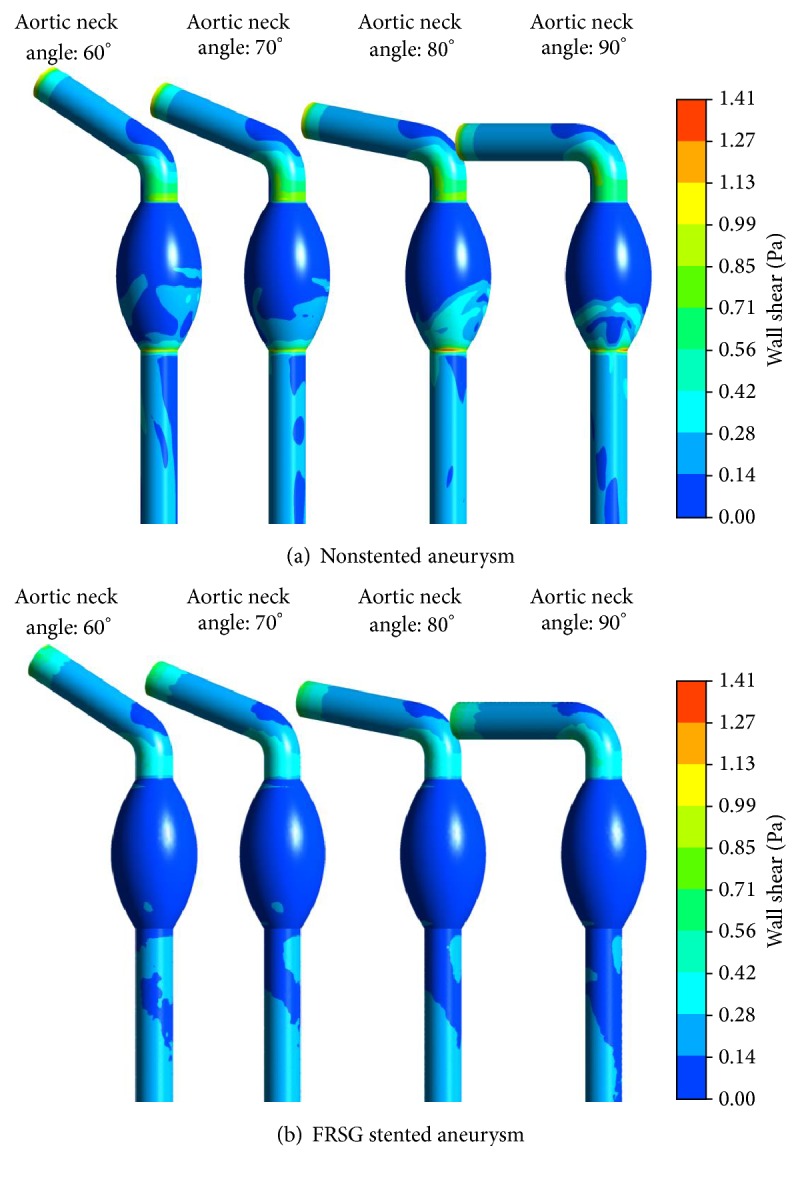
(a) Surface distribution of wall shear stress (WSS) of the nonstented aneurysm and (b) surface distribution of wall shear stress (WSS) of the FRSG stented aneurysm.

Figure 6 illustrates the surface distribution of wall shear stress (WSS) for the nonstented and stented aneurysm models. For nonstented models, thin strip of high velocity concentrated flow as observed in Figure 5 impinges on the proximal aortic neck and impacts the distal end of the aneurysm sac and thus created areas of high-wall shear stress. When FRSG is placed in the aneurysm, the flow is remodeled and shifted to the stent graft lumen. The elevated WSS in the aneurysm sac for nonstented models was significantly reduced by the implantation of FRSG. The strongest WSS reduction was located at the distal end of the aneurysm sac, since the area was impacted by high velocity concentrated flow in nonstented models. Additionally, areas that were not covered by the FRSG also exhibited minor reduction in WSS. Table 1 gives the average WSS in aneurysm sac (FRSG stented case) and shows that WSS was markedly reduced by 53%, 32%, and 37% for aortic neck angles of 60 degrees, 70 degrees, and 80 degrees, respectively. However, the percentage reduction of WSS for aortic neck angle of 90 degrees stood at 24%, which is lesser as compared to the rest of the cases.

### 3.3. Pressure Distribution

**Figure 7:**
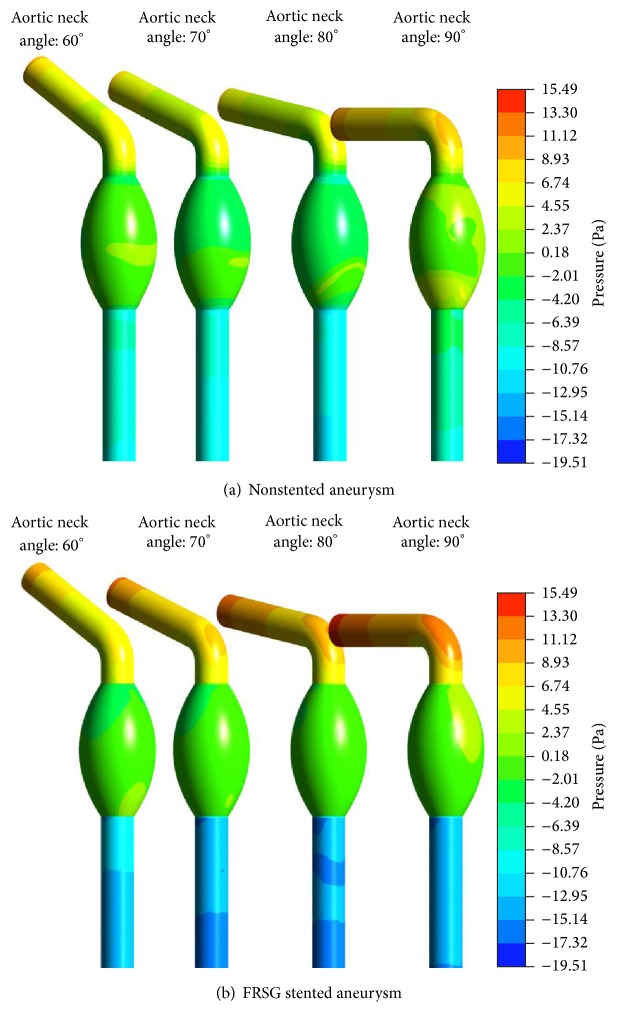
(a) Pressure distribution of the walls on the nonstented aneurysm and (b) pressure distribution of the walls on the FRSG stented aneurysm.

The pressure distribution at the aortic wall is shown in Figure 7. In the nonstented aneurysm, high-pressure region with maximum value of 5 Pa was observed at the distal end of the aneurysm sac for aortic neck angle of 90 degrees. By deploying FRSG into the aneurysm, the high-pressure regions at the aortic wall were reduced. In general observation, the angulation of the aortic neck does not have a significant effect on the pressure distribution in the aneurysm sac.

## 4. Discussion

Computational fluid dynamics simulation of nonstented model and stented model with proposed flow remodeling stent graft (FRSG) in highly angulated aortic aneurysm conditions was performed and the differences in terms of hemodynamic conditions were compared, respectively. Our study found that the aortic neck angles significantly influence the velocity flow field in nonstented models as the neck angles change the surface curvature between the aorta inlet and aneurysm, thus affecting the flow field downstream [17]. Our findings revealed that the increment of aortic neck angle resulted in the weakening of flow recirculation in the aneurysm sac and shifted the flow fields towards the center of the aorta. The simulation results confirmed that FRSG intervention significantly decreases average blood flow velocity in the aneurysm sac by at least 39%.

Our study revealed that the intervention of FRSG markedly reduces areas of high WSS located at the distal end of the aneurysm sac. The range of WSS percentage reduction obtained is similar to overlapping bare-metal stent studies [14]. Nonetheless, we would like to highlight that the improvement of WSS reduction for severely angulated aortic neck (90 degrees) is less. The minor improvement is due to the fact that the effect of high angulation has shifted the mainstream blood flow towards the center of aorta and thus lowers the initial WSS value in nonstented aneurysm. The proposed stent graft protects the area against the impact of high velocity concentrated flow and subsequently reduces the high-wall shear stress regions at the aortic wall. Therefore, the findings suggested that FRSG dampens the effect of direct impact of blood flow on the aneurysm wall by remodeling the mainstream flow into the stent graft lumen.

Several clinical studies employing flow diversion strategy using MFM stents [21–23] and MOUS [11–13] for the treatment of arterial aneurysms have reported short-term clinical success. Unlike conventional covered stent graft, both intervention strategies do not exclude blood flow into the aneurysm but rather act as a catalyst to allow thrombus formation and flow pattern modulation within the aneurysm sac. Our proposed flow remodeling stent graft (FRSG) uses the same modus operandi as MFM and MOUS. The porous design of the FRSG slows down the blood flow into the aneurysm sac, remodels the mainstream flow into the stent lumen, and reduces wall shear stress and pressure in the sac. It is important for the mainstream flow to be shifted into the stent lumen in order to decrease the overall blood flow velocities and reduce flow recirculation regions in the aneurysm sac. According to recent studies, untreated aneurysm was reported to rupture in the areas of flow recirculation region based on CFD investigation [24]. Therefore, the implantation of FRSG into the aneurysm eliminates erratic flow recirculation patterns and encourages the formation of uniform and laminar flow pattern in the aneurysm sac. The alteration of hemodynamic characteristics such as reduction of WSS and pressure at the aneurysm wall coupled with the combination of velocity reduction and laminar flow in the aneurysm provides a suitable environment for the formation of thrombus [14, 25]. The organized thrombus may effectively isolate the aneurysm and eventually lead to the resorption of aneurysm [26].

Hemodynamic studies of flow diversion stents in aortic aneurysm remain limited and development of flow diversion technology is still in its infancy. Thus, the gap should fuel interest of biomedical engineers and clinicians in this area. In this study, CFD simulation allows better understanding of the hemodynamic mechanisms pre- and post-FRSG intervention and could potentially help to optimize aneurysm treatment planning. Existing clinical studies showed that flow diversion concept decreases blood flow velocity within the aneurysm sac observed on fluoroscopy [12, 27]. Our simulation results support this observation and revealed more hemodynamic information such as wall shear stress and aneurysm flow patterns. The use of porous stent graft has also been studied in animal models showing organized thrombus formed within the aneurysm after implantation [28–30]. Based on the results of this study, the authors postulate that the flow diversion effect of FRSG provides a suitable hemodynamic environment for the formation of thrombus.

Endoleak remains the Achilles heel of conventional EVAR devices but the definition of endoleak for flow diversion devices should be modified due to the difference in mechanism of treatment [31]. The porous design of flow diversion device allows flow modulation in the aneurysm sac and not total aneurysm exclusion. Flow diversion device relies on the reduced blood flow to create a hemodynamic environment conducive for aneurysm thrombosis to happen naturally. Therefore, EVAR interventions using flow diversion devices typically regard type 1 endoleak as the main failure mode of treatment [32]. Type 1 endoleak is defined by the persistent filling of the aneurysm sac due to incomplete seal at either the proximal or the distal attachment site. In this study, the proposed FRSG is a fixed length stent graft with covered proximal and distal portions ensuring adequate seal zone while minimizing stent foreshortening issues. Therefore, we believe the risk of type 1 endoleak for the proposed FRSG is much lower and may offer a better proximal and distal seal zone.

Yet clinical practitioners remain cautious in adopting flow diversion devices such as MFM stents for the treatment of aortic aneurysm due to initial clinical studies that reported adverse outcome such as aneurysm rupture postoperative [33–35]. The authors would like to highlight that recent clinical experience showed that complications arise if the MFM intervention was performed outside the device indication for use [32]. Wrong device oversizing, device not landing in normal aorta, inadequate stent overlap, and poor selection of patients were the root cause of failed procedures [32]. Therefore, strict adherence to device indication for use and better understanding of device limitation can actually lead to good clinical outcomes [21]. However, it is worth mentioning that aneurysm sac size may increase and aneurysm thrombosis may take months to be completed [22, 31]. Therefore, patients are not immediately protected from aneurysm rupture with flow diversion device. As such, regular postoperative monitoring of aneurysm sac similar to conventional EVAR after surveillance should be practiced.

It is appropriate to point out that the findings of this study are limited by some simplification involved. Firstly, the present study is performed under steady state flow conditions. In real situation, blood flow is transient and pulsatile in nature, which varies with different phases during the cardiac cycle. The varying flow conditions can provide additional hemodynamic parameters such as oscillatory shear index (OSI) and relative residence time (RRT) [36, 37]. Studies have shown that high OSI is associated with aneurysm growth or rupture [38], while high RRT is linked to formation and growth of intraluminal thrombus [39]. These parameters are important to investigate since the concept of the flow remodeling stent graft provides an environment conducive to promote thrombus formation that will eventually lead to degradation of the aneurysm. Secondly, the idealized aortic aneurysm model studied in this study is simulated without branch arteries such as lumbar arteries and inferior mesenteric artery. Retrograde flow through branch vessels also known as endoleak type II can fill the aneurysm, thereby causing pressure to rise in the aneurysm sac [40]. An inclusion of branch arteries could elucidate the hemodynamic performance and flow behavior in the aneurysm sac under the influence of flow remodeling stent graft. Finally, the present study adopted the assumption of rigid wall used in the computational simulation. In the future, the compliance of aortic wall and stent graft can be considered in the simulation study as the flow-wall interaction will interfere with the wall displacement and thus alters the WSS pattern [41]. However, the consideration of running a fluid-structure interaction study is computationally extensive and may limit clinical adoption. Nonetheless, this current study may constitute the initial measure towards the development of novel stent graft device for the treatment of highly angulated aortic neck aneurysm.

## 5. Conclusion

In conclusion, computational fluid dynamics simulations have been performed to elucidate the effect of flow remodeling stent graft (FRSG) in complex abdominal aortic aneurysms with different aortic neck angulations. Similar to existing flow modulating based stents and overlapping bare-metal stents techniques, FRSG intervention impedes blood flow into the aneurysm sac, which significantly reduces the average flow velocity by 39% to 53%. By deploying FRSG into the aneurysm, blood flow is remodeled into the center of stent graft lumen and generates a laminar and uniform pattern in the aneurysm sac and hence eliminates erratic flow recirculation regions. FRSG intervention protects the aneurysm against the impacts of high velocity concentrated flow and decreases wall shear stress by more than 50%. As a result, the hemodynamic changes within the FRSG treated aneurysm may provide a favorable environment for the development of organized thrombus and eventually lead to resorption of the aneurysm. FRSG appears to be an alternative device for use in the treatment of aortic aneurysm and further in vitro and in vivo testing is warranted before clinical adoption.

## Figures and Tables

**Table 1:** Percentage average velocity magnitude and average wall shear stress (WSS) in the aneurysm sac of nonstented aneurysm and FRSG stented aneurysm.

Aortic neck angles	Average velocity in aneurysm sac (%)		Average WSS in aneurysm sac (%)	
	Nonstented	FRSG stented	Nonstented	FRSG stented
60°	100	59	100	47
70°	100	61	100	68
80°	100	46	100	63
90°	100	47	100	76

## References

[1] Clouse W. D., Hallett J. W., Schaff H. V., Gayari M. M., Ilstrup D. M., Melton L. J. (1998). Improved prognosis of thoracic aortic aneurysms. A population-based study. *The Journal of the American Medical Association*.

[2] Greenhalgh R. M., Brown L. C., Kwong G. P., Powell J. T., Thompson S. G. (2004). Comparison of endovascular aneurysm repair with open repair in patients with abdominal aortic aneurysm (EVAR trial 1), 30-day operative mortality results: randomised controlled trial. *The Lancet*.

[3] Prinssen M., Verhoeven E. L. G., Buth J. (2004). A randomized trial comparing conventional and endovascular repair of abdominal aortic aneurysms. *The New England Journal of Medicine*.

[4] Morrison T. M., Meyer C. A., Fillinger M. F. (2013). Eligiblity for endovascular repair of short neck abdominal aortic aneurysms. *Journal of Vascular Surgery*.

[5] Chocron S., Vaislic C., Kaili D., Bonneville J.-F. (2011). Multilayer stents in the treatment of thoraco-abdominal residual type B dissection. *Interactive Cardiovascular and Thoracic Surgery*.

[6] Benjelloun A., Henry M., Ghannam A. (2012). Endovascular treatment of a tuberculous thoracoabdominal aneurysm with the multilayer stent. *Journal of Endovascular Therapy*.

[7] Natrella M., Castagnola M., Navarretta F. (2012). Treatment of juxtarenal aortic aneurysm with the multilayer stent. *Journal of Endovascular Therapy*.

[8] Sultan S., Hynes N. (2015). Multilayer flow modulator stent technology: a treatment revolution for US patients?. *Expert Review of Medical Devices*.

[9] Biasetti J., Gasser T. C., Auer M., Hedin U., Labruto F. (2010). Hemodynamics of the normal aorta compared to fusiform and saccular abdominal aortic aneurysms with emphasis on a potential thrombus formation mechanism. *Annals of Biomedical Engineering*.

[10] Henry M., Polydorou A., Frid N. (2008). Treatment of renal artery aneurysm with the multilayer stent. *Journal of Endovascular Therapy*.

[11] Zhang Y., Lu Q., Zhao Z. (2014). Multiple overlapping uncovered stents as an alternative flow-diverting strategy in the management of peripheral and visceral aneurysms. *Journal of Vascular Surgery*.

[12] Zhang Y., Teng Z., Lu Q. (2014). Management of complicated aortic aneurysms using multiple overlapping uncovered stents: mid-term outcome from a cohort study. *Medicine*.

[13] Zhang L., Yin C.-P., Li H.-Y. (2013). Multiple overlapping bare stents for endovascular visceral aneurysm repair: a potential alternative endovascular strategy to multilayer stents. *Annals of Vascular Surgery*.

[14] Zhang P., Sun A., Zhan F., Luan J., Deng X. (2014). Hemodynamic study of overlapping bare-metal stents intervention to aortic aneurysm. *Journal of Biomechanics*.

[15] Wailliez C., Coussement G. CFD study of multilayer stent haemodynamics effects in abdominal aortic aneurysms.

[16] Sisto A., Restante A. L., Brik B. A., Preziosi L. Numerical simulation comparison between monolayer and multilayer flow modulator in a cerebral saccular aneurysm.

[17] Li Z., Kleinstreuer C. (2007). A comparison between different asymmetric abdominal aortic aneurysm morphologies employing computational fluid-structure interaction analysis. *European Journal of Mechanics—B/Fluids*.

[18] Fung Y. (2013). *Biomechanics: Circulation*.

[19] Egelhoff C. J., Budwig R. S., Elger D. F., Khraishi T. A., Johansen K. H. (1999). Model studies of the flow in abdominal aortic aneurysms during resting and exercise conditions. *Journal of Biomechanics*.

[20] Sheidaei A., Hunley S. C., Zeinali-Davarani S., Raguin L. G., Baek S. (2011). Simulation of abdominal aortic aneurysm growth with updating hemodynamic loads using a realistic geometry. *Medical Engineering and Physics*.

[21] Vaislic C. D., Fabiani J. N., Chocron S. (2014). One-year outcomes following repair of thoracoabdominal aneurysms with the multilayer flow modulator: report from the STRATO trial. *Journal of Endovascular Therapy*.

[22] Debing E., Aerden D., Gallala S., Vandenbroucke F., van den Brande P. (2014). Stenting complex aorta aneurysms with the cardiatis multilayer flow modulator: first impressions. *European Journal of Vascular and Endovascular Surgery*.

[23] Sultan S., Hynes N. (2013). One-year results of the multilayer flow modulator stent in the management of thoracoabdominal aortic aneurysms and type B dissections. *Journal of Endovascular Therapy*.

[24] Boyd A. J., Kuhn D. C. S., Lozowy R. J., Kulbisky G. P. (2015). Low wall shear stress predominates at sites of abdominal aortic aneurysm rupture. *Journal of Vascular Surgery*.

[25] Zhang P., Liu X., Sun A., Fan Y., Deng X. (2015). Hemodynamic insight into overlapping bare-metal stents strategy in the treatment of aortic aneurysm. *Journal of Biomechanics*.

[26] Sfyroeras G. S., Dalainas I., Giannakopoulos T. G., Antonopoulos K., Kakisis J. D., Liapis C. D. (2012). Flow-diverting stents for the treatment of arterial aneurysms. *Journal of Vascular Surgery*.

[27] Zhang Y., Lu Q., Pei Y. (2014). Total endovascular repair of thoracoabdominal aortic aneurysms with non-customized stent grafts. *Annals of Thoracic Surgery*.

[28] Nishi S., Nakayama Y., Ishibashi-Ueda H., Masato Y. (2014). Occlusion of canine aneurysms using microporous self-expanding stent grafts: long-term follow-up. *Clinical Neurology and Neurosurgery*.

[29] Nishi S., Nakayama Y., Ishibashi-Ueda H., Okamoto Y., Kinoshita Y. (2009). High-performance self-expanding stent graft: development and application to experimental aneurysms. *Journal of Artificial Organs*.

[30] Nishi S., Nakayama Y., Ishibashi-Ueda H., Matsuda T. (2003). Occlusion of experimental aneurysms with heparin-loaded, microporous stent grafts. *Neurosurgery*.

[31] Zhang Y.-X., Lu Q.-S., Feng J.-X. (2013). Endovascular management of pararenal aortic aneurysms with multiple overlapping uncovered stents. *Journal of Vascular Surgery*.

[32] Sultan S., Hynes N., Sultan M. (2014). When not to implant the multilayer flow modulator: lessons learned from application outside the indications for use in patients with thoracoabdominal pathologies. *Journal of Endovascular Therapy*.

[33] Lazaris A. M., Maheras A. N., Vasdekis S. N. (2012). A multilayer stent in the aorta may not seal the aneurysm, thereby leading to rupture. *Journal of Vascular Surgery*.

[34] Ferrero E., Gibello L., Ferri M., Viazzo A., Nessi F. (2014). Aortic arch rupture after multiple multilayer stent treatment of a thoracoabdominal aneurysm. *Journal of Vascular Surgery*.

[35] Cavalcante R. N., Nishinari K., Yazbek G., Krutman M., Bomfim G., Wolosker N. (2015). Severe visceral ischemia and death after multilayer stent deployment for the treatment of a thoracoabdominal aortic aneurysm. *Journal of Vascular Surgery*.

[36] Suh G.-Y., Les A. S., Tenforde A. S. (2011). Quantification of particle residence time in abdominal aortic aneurysms using magnetic resonance imaging and computational fluid dynamics. *Annals of Biomedical Engineering*.

[37] Les A. S., Shadden S. C., Figueroa C. A. (2010). Quantification of hemodynamics in abdominal aortic aneurysms during rest and exercise using magnetic resonance imaging and computational fluid dynamics. *Annals of Biomedical Engineering*.

[38] Xiang J., Natarajan S. K., Tremmel M. (2011). Hemodynamic-morphologic discriminants for intracranial aneurysm rupture. *Stroke*.

[39] Basciano C., Kleinstreuer C., Hyun S., Finol E. A. (2011). A relation between near-wall particle-hemodynamics and onset of thrombus formation in abdominal aortic aneurysms. *Annals of Biomedical Engineering*.

[40] Li Z., Kleinstreuer C. (2006). Computational analysis of type II endoleaks in a stented abdominal aortic aneurysm model. *Journal of Biomechanics*.

[41] Torii R., Oshima M., Kobayashi T., Takagi K., Tezduyar T. E. (2009). Fluid-structure interaction modeling of blood flow and cerebral aneurysm: significance of artery and aneurysm shapes. *Computer Methods in Applied Mechanics and Engineering*.

